# Transforming Growth Factor Beta 1 Alters Glucose Uptake but Not Insulin Signalling in Human Primary Myotubes From Women With and Without Polycystic Ovary Syndrome

**DOI:** 10.3389/fendo.2021.732338

**Published:** 2021-10-11

**Authors:** Luke C. McIlvenna, Rhiannon K. Patten, Andrew J. McAinch, Raymond J. Rodgers, Nigel K. Stepto, Alba Moreno-Asso

**Affiliations:** ^1^ Institute for Health and Sport, Victoria University, Melbourne, VIC, Australia; ^2^ Australian Institute for Musculoskeletal Science (AIMSS), Victoria University, Melbourne, VIC, Australia; ^3^ Discipline of Obstetrics and Gynaecology, School of Medicine, Robinson Research Institute, The University of Adelaide, Adelaide, SA, Australia

**Keywords:** Extracellular matrix, fibrosis, insulin resistance, skeletal muscle, endocrinology, cytokines

## Abstract

Women with polycystic ovary syndrome (PCOS), commonly have profound skeletal muscle insulin resistance which can worsen other clinical features. The heterogeneity of the condition has made it challenging to identify the precise mechanisms that cause this insulin resistance. A possible explanation for the underlying insulin resistance may be the dysregulation of Transforming Growth Factor-beta (TGFβ) signalling. TGFβ signalling contributes to the remodelling of reproductive and hepatic tissues in women with PCOS. Given the systemic nature of TGFβ signalling and its role in skeletal muscle homeostasis, it may be possible that these adverse effects extend to other peripheral tissues. We aimed to determine if TGFβ1 could negatively regulate glucose uptake and insulin signalling in skeletal muscle of women with PCOS. We show that both myotubes from women with PCOS and healthy women displayed an increase in glucose uptake, independent of changes in insulin signalling, following short term (16 hr) TGFβ1 treatment. This increase occurred despite pro-fibrotic signalling increasing *via* SMAD3 and connective tissue growth factor in both groups following treatment with TGFβ1. Collectively, our findings show that short-term treatment with TGFβ1 does not appear to influence insulin signalling or promote insulin resistance in myotubes. These findings suggest that aberrant TGFβ signalling is unlikely to directly contribute to skeletal muscle insulin resistance in women with PCOS in the short term but does not rule out indirect or longer-term effects.

## Introduction

Polycystic ovary syndrome (PCOS) is a common endocrine condition that affects 8-13% of women of reproductive age, with health implications throughout the lifespan (1, 2). The condition can be characterised by androgen excess, ovulatory dysfunction and polycystic ovaries (3), with a combination of at least two of these features required to meet the diagnostic criteria (4). Traditionally, PCOS has been considered a reproductive condition based upon the diagnostic criteria; however, insulin resistance appears to be a key feature and a driver of the symptoms of PCOS, with approximately 38-80% of women with PCOS being insulin resistant when measured by euglycaemic–hyperinsulinaemic clamp (5, 6). The insulin resistance and subsequent hyperinsulinaemia appear to contribute to hyperandrogenism, ovulatory dysfunction and subfertility, highlighting the importance of understanding the mechanisms by which insulin resistance develops in women with PCOS.

Several insulin signalling defects have been observed *in vivo* and *in vitro* in skeletal muscle of women with PCOS. Distinctly in PCOS, there appear to be intrinsic defects in the post-binding insulin receptor signalling, present in the absence of obesity or reduced glucose tolerance (7–10). Contrary, other studies were not able to identify any defects in skeletal muscle insulin signalling during euglycaemic-hyperinsulinaemic clamps or insulin stimulation in myotubes despite the given population of overweight and lean women with PCOS having severe insulin resistance (11–14). Taken together, this suggests that environmental factors found in the circulation may play a more significant role than intrinsic defects in the development of PCOS-specific insulin resistance.

Given the heterogeneity of PCOS, multiple circulating factors could lead to the development of insulin resistance. A possible candidate involved in the development of metabolic abnormalities in women with PCOS is transforming growth factor-beta (TGFβ) ligands. It has been identified that several of the TGFβ ligands play a role in the pathophysiology of PCOS. These TGFβ ligands are responsible for alterations in ovarian hormones and morphology, with the thickening of the ovarian capsule and stroma caused by an increase in collagen deposition and fibrotic tissue related to a dysregulation of TGFβ superfamily ligands (15–17). The sustained activation of SMAD2/3 signalling by TGFβ1 leads to an increase in collagen production and fibrotic factors, causing extracellular matrix (ECM) remodelling (18, 19). In particular, TGFβ1 can upregulate the pro-fibrotic cytokine connective tissue growth factor (CTGF/CCN2) in skeletal muscle cells and tissue (20, 21),. And subsequently, promote production of collagen and other fibrotic proteins such as fibronectin and alpha-SMA (22–24).

TGFβ ligands, including TGFβ1, are elevated in the serum of women with PCOS (25–28). This evidence presents the possibility that TGFβ1 exerts effects beyond the reproductive tissues and contributes to other pathophysiological features in women with PCOS. However, the interaction between TGFβ signalling, ECM and insulin resistance is not fully understood. Dysregulation of ECM remodelling *via* elevated TGFβ ligands and inflammation results in excess collagen deposition, which may create a physical barrier that could impair the uptake of glucose and the binding of insulin to the receptor, contributing to the development of insulin resistance (29–32). In line with these findings, gene expression of key components of the TGFβ signalling pathway is altered in the skeletal muscle of women with PCOS (33–35). An increase in glucose and insulin can promote the translocation of intracellular TGFβ receptors to the cell surface in a variety of cell types (36, 37). This occurs *via* the activation of Akt and is regulated by subsequent activation of AS160 to enhance TGFβ responsiveness (36). This translocation of TGFβ receptors causes an amplification of TGFβ signalling through SMAD activation (38). The interaction between insulin and TGFβ signalling may be particularly pertinent in relation to women with PCOS, given that many have hyperinsulinemia.

TGFβ signalling *via* SMAD-Akt-mTOR pathway may be responsible for insulin resistance in women with PCOS (33, 39). This is due to SMAD signalling, causing perturbations in Akt and mTOR signalling (40, 41). The interaction of TGFβ1 with MTORC1 (mTOR and raptor) and MTORC2 (mTOR and rictor), has been explored in other cell types and conditions focusing on pulmonary fibrosis (42–45). These studies show that TGFβ1 activation of SMAD3 leads to an increase in collagen synthesis *via* mTOR signalling. This excess collagen synthesis is coupled with metabolic disturbances and an increase in glycolysis. In support of the metabolic role of TGFβ1 signalling, SMAD3 knockout mice are protected from diet-induced obesity and diabetes (46, 47). Whether or not these effects would occur in the skeletal muscle remains to be determined.

Hyperglycaemia and hyperinsulinemia, two significant features of PCOS pathophysiology, can lead to rapid translocation of intracellular TGFβ receptors 1 and 2 to the cell surface in various cell types (36, 37, 48). This translocation of TGFβ receptors causes an amplification of TGFβ signalling through SMAD activation (38), leading to an increase in cell migration (36, 49). This process is capable of stimulating oxidative stress and adverse ECM remodelling. Collectively, this presents the possibility that aberrant TGFβ signalling could induce insulin resistance *via* direct dysregulation of insulin signalling or by negatively regulating the function and composition of the skeletal muscle ECM. Therefore, this study aimed to determine the role of TGFβ1 in metabolic dysfunction and to generate a greater understanding of the aetiology of PCOS.

## Materials And Methods

### Participants

Seven overweight women with PCOS and seven lean healthy women participated in the study. Participants’ clinical characteristics are shown in **Table 1**. PCOS was diagnosed using the Rotterdam Criteria (4), with the diagnosis being confirmed by an endocrinologist. Rotterdam criteria required confirmation of two of the following: (i) oligo- or anovulation; (ii) clinical (hirsutism and acne) and/or biochemical hyperandrogenism; (iii) polycystic ovaries on ultrasound and the exclusion of other causes of hyperandrogenism. The healthy control group consisted of women without any features of PCOS. All women were Caucasian, premenopausal and aged between 18-45 yr. The exclusion criteria for both groups included menopause, pregnancy, smoking, type 1 or type 2 diabetes mellitus, uncontrolled hypertension (> 160/100 mm/Hg), cardiac ischemia, established cardiovascular disease, renal impairment and malignancy, and use of medications that interfere with endpoints (e.g., oral contraception, insulin-sensitisers, anti-androgens, progestins, anti-hypertensives, and lipid-lowering agents). Ethical approval was obtained from the Victoria University Human Research Ethics Committee (Reference - HRE17-232). All participants provided written informed consent prior to participation in the study.

**Table 1:** Clinical Characteristics.

	Healthy Women (n = 7)	Women with PCOS (n = 7)
***General characteristics***		
**Age**	26 ± 2	30 ± 2
**Weight (kg)**	65 ± 5^b^	99 ± 7
**BMI (kg/m^2^)**	22.1 ± 1.0^b^	36.7 ± 2.5
**Lean mass (%)**	68 ± 2^b^	49 ± 2
**Fat mass (%)**	30 ± 2^b^	49 ± 3
**PCOS Phenotype**	N/A	A = 3 B = 2 C = 0 D = 2
***Fasting blood measurements***		
**Free Testosterone (pmol/L)**	13.54 ± 2.44^a^	36.9 ± 5.45
**Total Testosterone (nmol/L)**	0.92 ± 0.12^a^	1.48 ± 0.14
**Insulin (µIU/mL)**	9.9 ± 0.9^a^	15.6 ± 2.4
**Glucose (mM)**	4.4 ± 0.1	4.7 ± 0.1
**HBA1C (%)**	5.13 ± 0.05	5.19 ± 0.19
**SHBG (nmol/L)**	54.1 ± 10.0	30.7 ± 8.0
**Dihydrotestosterone (nmol/L)**	0.33 ± 0.07	0.33 ± 0.01
**Estradiol (pmol/L)**	179.9 ± 76.1	284.9 ± 62.0
**Androstenedione (nmol/L)**	3.22 ± 0.34^a^	5.14 ± 0.58
**ALT (IU/L)**	21.0 ± 4.6	45.9 ± 12.4
**AST (IU/L)**	22.1 ± 2.2	26.6 ± 3.9
**Cholesterol (mmol/L)**	3.90 ± 0.27^a^	5.13 ± 0.36
**Triglycerides (mmol/L)**	0.6 ± 0.1^a^	1.4 ± 0.1
**HDL (mmol/L)**	1.57 ± 0.14^a^	2.85 ± 0.48
**LDL (mmol/L)**	2.0 ± 0.2	2.7 ± 0.1
***Euglycaemic-hyperinsulinaemic clamp***		
**GIR (mg/lbmkg/min)**	16.36 ± 1.9^b^	6.75 ± 1.1
**Insulin sensitivity index**	14.97 ± 1.7^a^	7.0 ± 1.0

Data presented as mean (± SEM). PCOS phenotypes defined according to the Rotterdam Criteria, with the following features present in each phenotype as follows: A = Hyperandrogenism, ovulatory dysfunction and polycystic ovarian morphology. B = Hyperandrogenism and ovulatory dysfunction. C = Hyperandrogenism and polycystic ovarian morphology D = Ovulatory dysfunction and polycystic ovarian morphology. SHBG, Sex hormone-binding globulin; AST, aspartate aminotransferase; ALT, alanine aminotransferase; HDL, high-density lipoproteins; lbmkg, lean body mass in kilograms; LDL, low-density lipoproteins; GIR, Glucose infusion rate. Insulin sensitivity index calculated using the following formula: (Glucose infusion rate/lean body mass)/Steady state insulin)*100. ^a^P < 0.05 vs. PCOS. ^b^P < 0.001 vs. PCOS.

### *In Vivo* Data Collection

Participants reported to the lab following an overnight fast, resting blood samples were collected, and a euglycaemic–hyperinsulinaemic clamp was performed to determine insulin sensitivity following methods as previously described (50, 51). Analysis of fasted blood samples for insulin (Human Insulin-Specific RIA, HI-14K, Millipore) and AMH (Ultra-Sensitive AMH/MIS ELISA, AL-105, Anash Labs) was carried out at Victoria University, with other measures being carried out by an accredited pathology lab at Monash pathology, Australia. For further details regarding blood sample collection and analysis see (52):. Lean mass and fat mass were estimated by a whole-body dual-energy X-ray absorptiometry (DXA) scan (GE Lunar Prodigy, GE Lunar Corp, Madison WI, USA; operating system version 9).

### Cell Culture

Following an overnight fast, a muscle biopsy was obtained from the *vastus lateralis* of each participant using the modified Bergstrom technique with suction (53, 54). Following collection, approximately 40-50 mg of muscle was minced into small pieces (< 1-2 mm^3^) and enzymatically disassociated with 0.05% Trypsin-EDTA (Gibco, Thermo-fisher, Melbourne, Australia) on an orbital shaker for 20 min at room temperature. This process was repeated twice with a total end volume of 45 ml of cell suspension. Five ml of fetal bovine serum (Gibco, Thermo-fisher, Melbourne, Australia) was then added to inactivate the trypsin. The cell suspension was filtered through 100 µm cell strainer (Falcon, Thermo-fisher, Melbourne, Australia) to remove any undigested tissue and then centrifuged for 10 min at 1500 rpm. Cells were resuspended in α-MEM (Gibco, Thermo-fisher, Melbourne, Australia) containing 5.5 mM glucose, supplemented with 10% fetal bovine serum, 0.5% penicillin-streptomycin (Sigma Aldrich, St Lewis, MO, USA) and 0.5% amphotericin B (Sigma Aldrich, St Lewis, MO, USA), plated into a T25 flask (Greiner Bio-one, Frickenhausen, Germany) previously coated with ECM gel (Geltrex™ LDEV-Free Reduced Growth Factor Basement Membrane Matrix, Thermo-fisher, Melbourne, Australia) and cultured at 37°C in 5% CO_2._ Growth medium was changed after 24 h and then every second day thereafter. For further details of methods used, see Cornall etal. (55).

After reaching 70-80% confluence, satellite cells were purified following the method by Agley etal. (56). Magnetic activated cell sorting (MACS) with anti-CD56 microbeads (MACS #130-050-401, Miltenyi Biotec, Bergisch Gladbach, Germany) and MS column (MACS, #130-091-632, Miltenyi Biotec, Bergisch Gladbach, Germany) were used in order to separate a fraction of enriched myogenic cells (CD56+). This enriched fraction of satellite cells was then plated into ECM-coated T75 flasks. After reaching 80-90% confluence, myoblasts were plated for experiments (passage 4) into 6-well plates for protein expression studies and 12-well plates for glucose uptake experiments. Growth medium was changed every second day until cells reached 80-90% confluence, then differentiation was started. Differentiation medium (normal-glucose α-MEM supplemented with, 2% horse serum, 0.5% penicillin-streptomycin and 0.5% amphotericin B) was added to the cells and changed every day for five days. Differentiated myotubes were then exposed for 16 h to TGFβ1 (1 ng/ml and 5 ng/ml) (Transforming Growth Factor-β1 human, T7039, Sigma Aldrich, St Lewis, MO, USA), following treatment with TGFβ1 myotubes were treated with or without insulin (100 nM) (ActRapid, Human Insulin, NovoNordisk ﻿Bagsvaerd, Denmark) for 30 min. An untreated control condition with no TGFβ1 treatment was also included. Treatments and control conditions were prepared in serum-free low glucose media (α-MEM with 0.1% BSA, 0.5% penicillin-streptomycin and 0.5% amphotericin B).

### Glucose Uptake

A radioactivity-based assay utilising [2-^3^H]deoxy-D-glucose ([2-^3^H]DG) (PerkinElmer, NET238C001MC, Waltham, MA, USA) was used to measure glucose uptake (57). Myotubes were pre-incubated overnight (16 hours) in serum-free media with or without the previously described treatments of TGFβ1, Cells were washed three-times and pre-incubated with Krebs buffer (10 mM HEPES, 2.5 mM NaH_2_PO_4_, 150 mM NaCl, 5 mM KCl, 1.2 mM CaCl_2_, 1.2 mM MgSO_4_, 0.1% BSA) with and without insulin (100 nM) for 30 minutes. The pre-incubation with Krebs buffer was used to deplete pre-existing glucose. A condition with 50 μM of Cytochalasin B was included as a negative control to determine non-specific glucose uptake through diffusion. To assess glucose uptake, 10 μM of 2-deoxy-D-glucose containing 1 μCi/mL/well ([2-^3^H]DG) was added for 15 min at 37°C. The use of modified radiolabelled glucose means the glucose cannot be metabolized and accumulates in the cell. This allows glucose uptake to be quantified by the radioactivity detected from the labelled glucose within the cell. The cells were then rinsed four times with cold PBS and lysed in 500 μl of 0.1 M NaOH. Four hundred microlitres of the lysate were then transferred to scintillation vials, and 100 µl was retained for subsequent total protein quantification. Glucose uptake was determined using a liquid scintillation counter (Tri-Carb 2910 TR, Perkin-Elmer, IL, USA), with the unit of measurement in picomoles of [2-^3^H]DG taken up per min per mg of total protein.

### Western Blotting

Cell lysates were prepared using ice-cold RIPA buffer (Product No.89900, ThermoFisher Sci Waltham, MA, USA) with the addition of a phosphatase and protease inhibitor cocktail at 1:100 (Halt™ Phosphatase Inhibitor Cocktail Product No.78440, ThermoFisher Sci, Melbourne, Australia) (55). The total protein concentration for each sample was determined using Red 660 protein assay (cat no. 786-676, G-Biosciences, St Louis, Missouri, USA) with SDS Neutralizer (cat no. 786-673, G-Biosciences, St Louis, Missouri, USA). A total of 50 µg of each sample was electrophoresed on 10% Criterion™ TGX Stain-Free™ protein gels (Bio-rad, #5678034, Gladesville, NSW, Australia) for 90 min at 200 V. Following separation by gel electrophoresis, and proteins were then transferred to a nitrocellulose membrane (Bio-rad, #1704271, Gladesville, NSW, Australia). The transfer was performed using Trans-Blot^®^ Turbo™ Transfer System (Bio-rad, #1704150, Gladesville, NSW, Australia) using the following protocol: 2.5 A, 25 V, for 7 min. The membrane was then imaged for total protein using the stain-free protocol on ChemiDoc™ XRS+ System (Bio-rad #1708265, Gladesville, NSW, Australia). All membranes were blocked for 1 h in 5% skim milk in Tris-buffered saline (TBS) plus 0.1% Tween 20 (TBS-T). Membranes were then washed for 3 x 5 min in TBS-T, then incubated overnight on a rocking platform at 4°C in primary antibody solution containing selected antibody **(** **Table 2**). The next day, membranes were washed for 3 x 5 min in TBS-T then incubated in appropriate secondary horseradish peroxidase-conjugated antibody (1:10,000) for 90 min. Proteins were visualised by ultra-sensitive enhanced chemiluminescence (SuperSignal™ West Femto Maximum Sensitivity Substrate, Thermo Scientific, #34094, ﻿Melbourne, Australia). Images of membranes for total protein and target proteins were analysed using Bio-rad Image Lab 6.0.1 (Bio-rad, Gladesville, NSW, Australia) to determine band density. The band density data were normalised to total protein content for each lane and to the internal standard loaded in each gel using the stain-free method (58, 59).

**Table 2:** Antibody list.

Antibody	Company	Concentration
Phospho-Smad3 (Ser423/425) (C25A9) (9520S)	Cell Signalling	1:1000
Smad3 (C67H9) (9523S)	Cell Signalling	1:1000
Phospho-Smad1 (Ser463/465)/Smad5 (Ser463/465)/Smad9 (Ser465/467) (D5B10) (13820S)	Cell Signalling	1:1000
Smad5 (D4G2) (12534S)	Cell Signalling	1:1000
Smad4 (D3M6U) (38454S)	Cell Signalling	1:1000
IRS1 (Phospho s312) (ab4865)	Abcam	1:1000
IRS-1 (D23G12) (3407S)	Cell Signalling	1:1000
Phospho-Akt (Ser473) (D9E) (4060)	Cell Signalling	1:2000
Akt (pan) (C67E7)	Cell Signalling	1:1000
PI3 Kinase p85-alpha (6G10) (13666S)	Cell Signalling	1:1000
PI3 Kinase p110-alpha (C73F8) (4249S)	Cell Signalling	1:1000
phospho-AS160 (Thr642) (4288)	Cell Signalling	1:1000
AS160 (C69A7) (2670)	Cell Signalling	1:1000
CTGF (D8Z8U) (86641)	Cell Signalling	1:1000
Glucose transporter 4 (ab654)	Abcam	1:4000
Glucose Transporter GLUT1 [SPM498] (ab40084)	Abcam	1:2000
Collagen III antibody [EPR17673] (ab184993)	Abcam	1:1000
Anti-Collagen I antibody (ab34710)	Abcam	1:1000
Phospho-mTOR (Ser2448) (2971S)	Cell Signalling	1:1000
mTOR (2972S)	Cell Signalling	1:1000
Phospho-Raptor (Ser792) (2083S)	Cell Signalling	1:1000
Raptor (24C12) (2280S)	Cell Signalling	1:1000
Phospho-Rictor (Thr1135) (D30A3) (3806S)	Cell Signalling	1:1000
Rictor (53A2) (2114S)	Cell Signalling	1:1000
Secondary Anti-rabbit IgG, HRP-linked Antibody (7074S)	Cell Signalling	1:10000
Secondary Pierce Anti-Mouse IgG (Goat) - HRP-Labelled (31430)	Thermofisher	1:10000

### Statistical Analysis

All analyses were carried out using SPSS (Version 26, IBM), and figures were created using GraphPad Prism Version 8 (GraphPad Software Inc., San Diego, USA). All data are reported as mean ± standard error of the mean (SEM) unless stated otherwise, and statistical significance was declared when P < 0.05. Clinical characteristics of groups were compared with two-tailed unpaired Student’s t-test. Statistical analysis for glucose uptake and protein expression were determined by two-way repeated measures ANOVA with Fisher’s least significant difference multiple comparisons post-hoc. To detect any outliers in the data, studentised residuals were assessed. The distributions of the data were tested using the Shapiro-Wilk test when data were not normally distributed; a log transformation was applied.

## Results

### Clinical Characteristics

Women with PCOS had greater body weight, BMI, fat mass and less lean mass than healthy women (P < 0.001) (**Table 1**). Consistently, women with PCOS displayed features of hyperandrogenism with greater levels of free and total testosterone and androstenedione compared to healthy women (P < 0.05) (**Table 1**), reflective of the main features of PCOS. There were no differences between the groups for the other reproductive hormones measured: SHBG, dihydrotestosterone, and estradiol (P > 0.05). Women with PCOS displayed profound insulin resistance with higher levels of fasting insulin and a ~50% reduction in insulin sensitivity as measured by euglycaemic–hyperinsulinaemic clamp (P < 0.05) (**Table 1**). Both groups had normal glucose levels and markers of liver health: ALT and AST (P > 0.05) (**Table 1**). While women with PCOS displayed elevated levels of cholesterol, triglycerides, HDL, these remained within reference ranges (P < 0.05) (**Table 1**).

### SMAD Signalling

**Figure 1:**
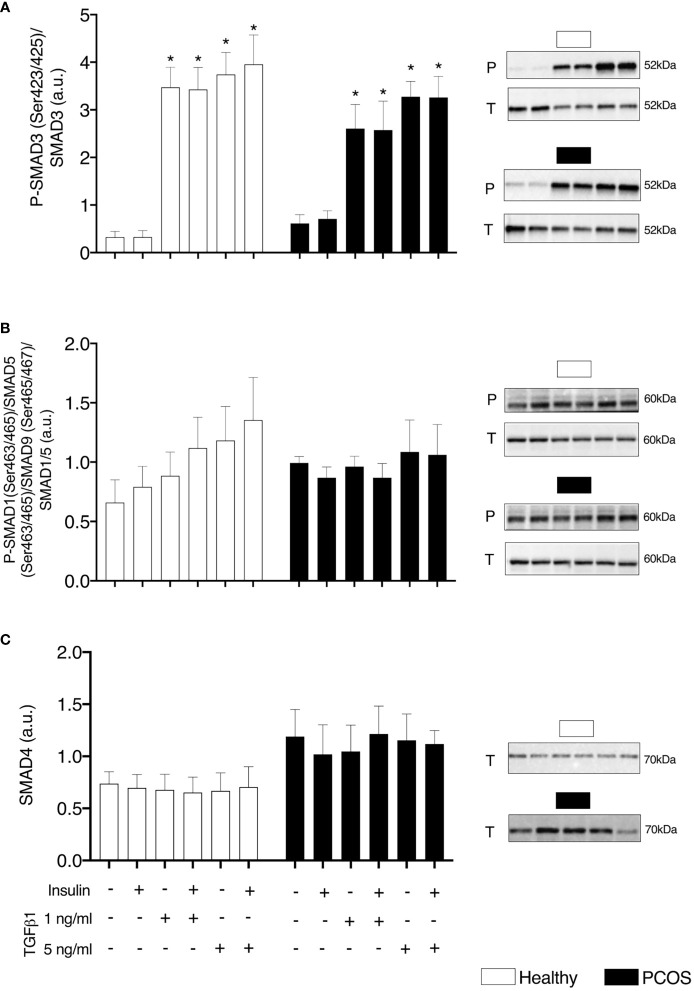
TGFβ Signalling. **(A)** SMAD3 phosphorylation relative to total SMAD3 expression, **(B)** SMAD1/5/8 phosphorylation relative to total SMAD1/5 expression, and **(C)** Total SMAD4 expression, following 16 h with no treatment or with TGFβ1 (1 ng/ml or 5 ng/ml) in a basal or insulin (100 nM) stimulated state. Data are reported as Mean ± SEM. AU, arbitrary units (defined as band density values). *significantly different from untreated control of the respective group (P < 0.05). Phospho (P) and Total (T). Healthy: N = 5 participants (clear bars), PCOS: N= 5 participants (filled bars).

There were no differences in pSMAD3/SMAD3 between groups (P > 0.05, **Figure 1A**). There was a main effect of treatment for pSMAD3/SMAD3 (P < 0.001, **Figure 1B**). Following treatment with TGFβ1 (1 ng/ml and 5 ng/ml) with and without insulin, there was an increase in pSMAD3/SMAD3 in myotubes from both healthy women and women with PCOS (1 ng/ml ± insulin: P < 0.001, 5 ng/ml ± insulin: P < 0.001, **Figure 1B**). We confirmed that TGFβ1 did not alter the expression of SMAD 4 in either group (P > 0.05, **Figure 1C**) and did not activate pSMAD1/5/9/SMAD5 signalling (P > 0.05, **Figure 1B**) associated with an anti-fibrotic response, contrary to the action of SMAD3 signalling.

### Glucose Uptake

**Figure 2:**
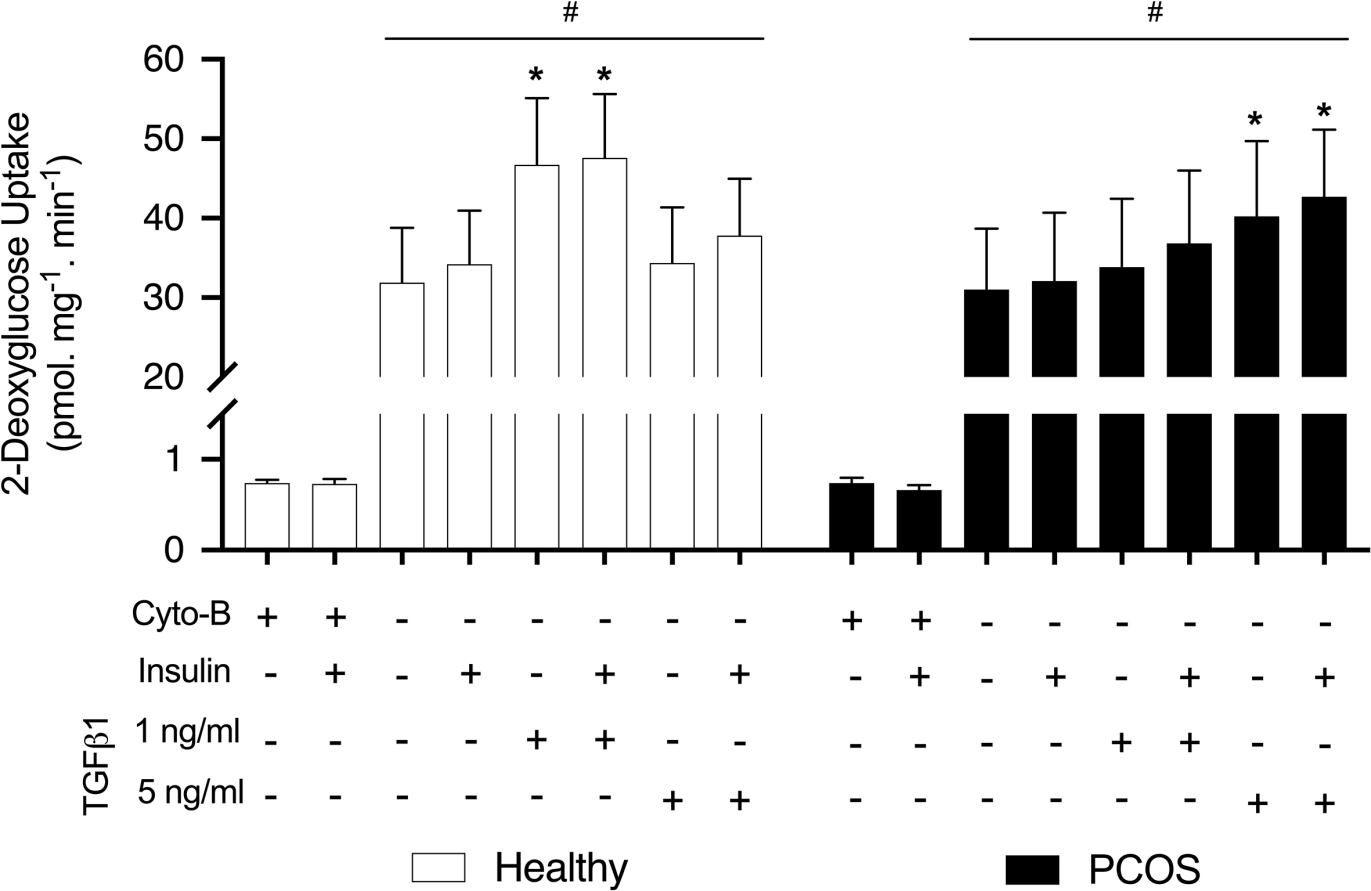
Glucose uptake. [2-^3^H] deoxy-D-glucose uptake in myotubes from women with and without PCOS. Differentiated myotubes were treated with TGFβ1 (1 ng/ml or 5 ng/ml) or untreated as a control condition for 16 hours, and this was followed by 30 min incubation with or without insulin (100 nM) to allow for the assessment of basal and insulin stimulated glucose uptake. A condition with 50 µM Cytochalasin B (Cyto-B) was used to determine the contribution of non-transporter mediated glucose uptake. Data are reported as Mean ± SEM. *significantly different from untreated control of the respective group (P < 0.05). ^#^significantly different from Cyto-B and Cyto-B + insulin. Healthy: N = 7 participants (clear bars), PCOS: N= 7 participants (filled bars).

In order to establish the effects of TGFβ1 on insulin sensitivity and glucose transport we performed a glucose uptake assay using [2-^3^H]DG. Both groups displayed similar responses for basal and insulin-stimulated glucose uptake in the untreated condition (P > 0.05, **Figure 2**). There was a significant effect of TGFβ1 treatment with and without insulin in both groups (P < 0.01, **Figure 2**). In particular, a significant increase in glucose uptake was observed following treatment with 1 ng/ml of TGFβ1 with and without insulin in the myotubes from healthy women (P < 0.001, **Figure 2**) and 5 ng/ml of TGFβ1 with and without insulin in myotubes from women with PCOS (P < 0.005) (**Figure 2**). In addition, we observed that Cytochalasin B inhibited glucose uptake by ~97%, confirming that the vast majority of the glucose uptake in the myotubes was indeed occurring through the glucose transporters.

### Insulin Signalling

**Figure 3:**
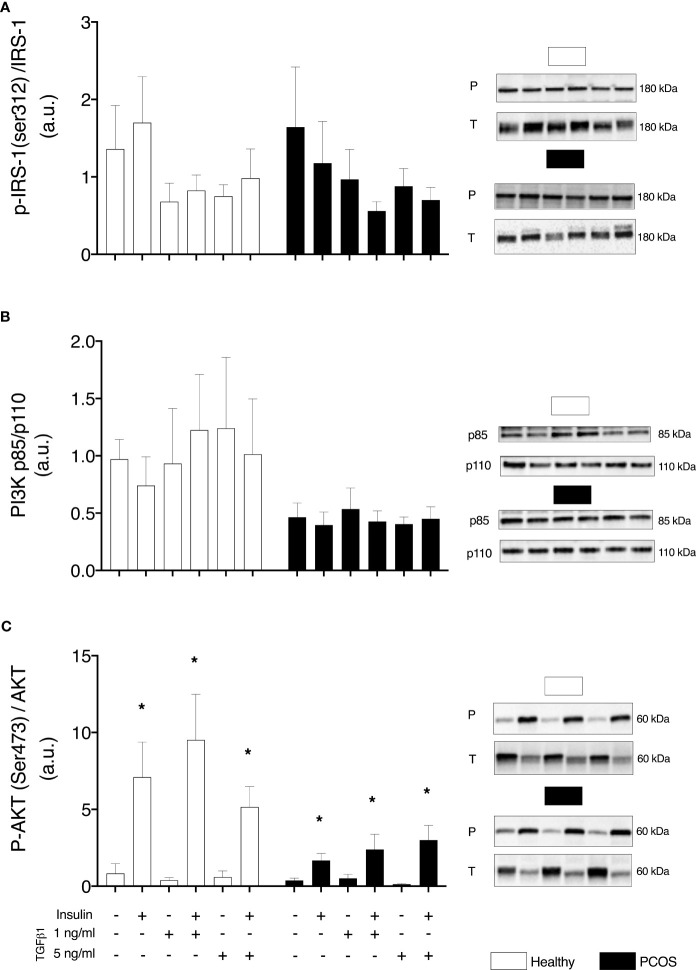
Insulin signalling. **(A)** IRS-1 phosphorylation relative to total IRS-1 expression. **(B)** Total protein expression PI3K-p85/PI3K p110 ratio. **(C)** AKT phosphorylation relative to total AKT expression, following 16 h with no treatment or with TGFb1 (1 ng/ml or 5 ng/ml) and 30 min insulin stimulation (0 nM or 100 nM). Data are reported as Mean ± SEM. AU, arbitrary units (defined as band density values). *significantly different from untreated control of the respective group (P < 0.05). Phospho (P) and Total (T). Healthy: N = 5 participants (clear bars), PCOS: N= 5 participants (filled bars).

We did not observe any differences in the proximal insulin receptor signalling, pIRS-1/IRS-1, between groups (P > 0.05, **Figure 3A**) or following treatment with TGFβ1 (P > 0.05, **Figure 3A**). Similarly, there were no differences in PI3K p85/PI3K p110 expression between groups (P > 0.05, **Figure 3B**) or following treatment (P > 0.05, **Figure 3B**). Despite not detecting any differences in pAkt/Akt between groups (P > 0.05, **Figure 3C**), there was a main effect of treatment (P < 0.001). An increase in the expression of pAkt/Akt was observed in myotubes from healthy women and women with PCOS following treatment with insulin (healthy: P < 0.01, PCOS: P < 0.001, **Figure 3C**). In addition, pAkt/Akt was also increased in both groups following the treatment with TGFβ1 1 ng/ml with insulin (healthy: P < 0.01, PCOS: P < 0.001, **Figure 3B**) and TGFβ1 5 ng/ml with insulin (healthy: P < 0.05, PCOS: P < 0.001, **Figure 3B**).

### mTOR Signalling

**Figure 4:**
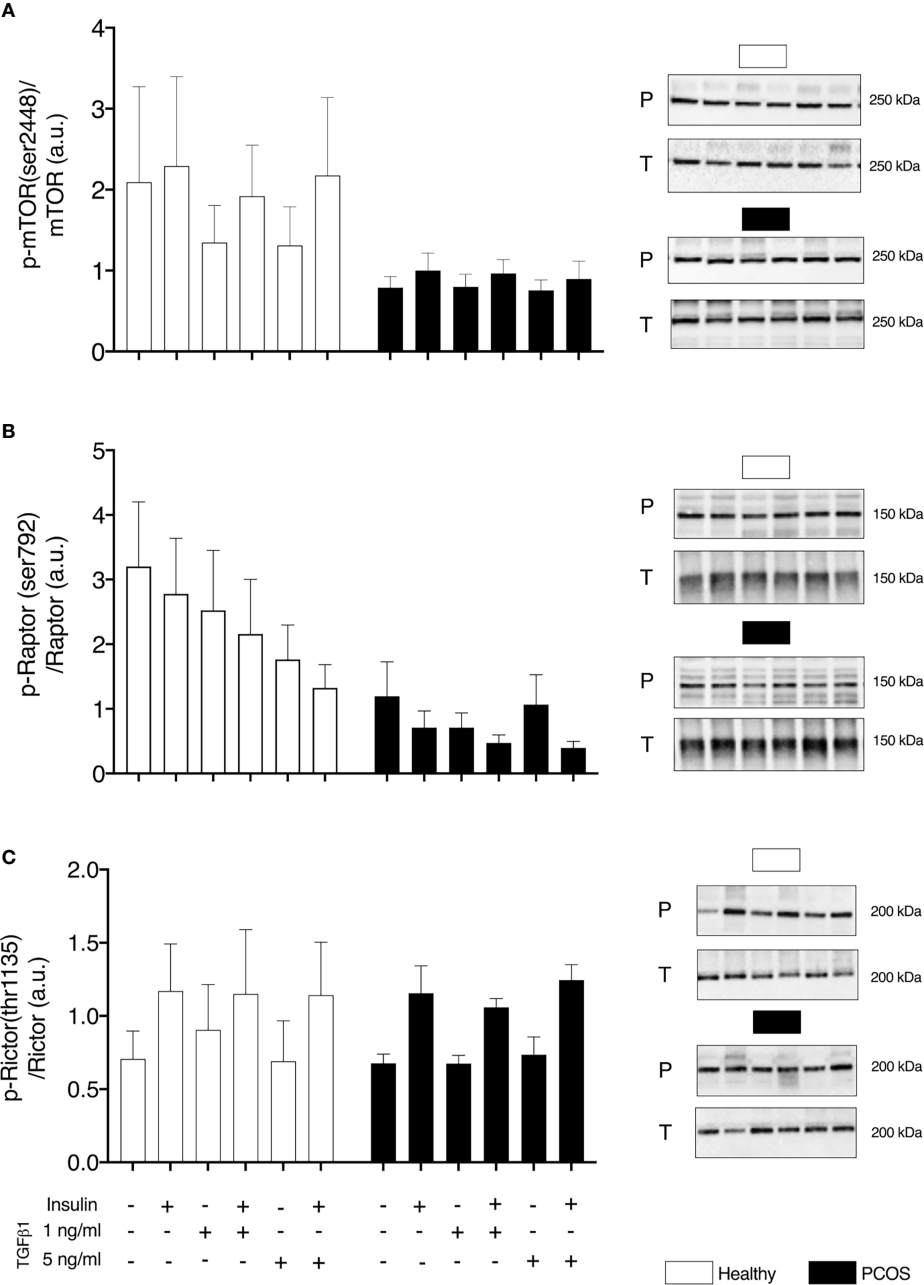
mTOR signalling. **(A)** mTOR phosphorylation relative to total mTOR expression. **(B)** Raptor phosphorylation relative to total Raptor expression. **(C)** Rictor phosphorylation relative to total Rictor expression, following 16 h with no treatment or with TGFβ1 (1 ng/ml or 5 ng/ml) and 30 min insulin stimulation (0 nM or 100 nM). Data are reported as Mean ± SEM. AU, arbitrary units (defined as band density values). Phospho (P) and Total (T). Healthy: N = 5 participants (clear bars), PCOS: N= 5 participants (filled bars).

In order to determine if upregulated SMAD signalling interferes with mTOR signalling, we assessed the phosphorylation of mTOR and its complexes. No group differences were identified for pmTOR/mTOR (P > 0.05, **Figure 4A**), pRaptor/Raptor (P > 0.05, **Figure 4B**) or pRictor/Rictor (P > 0.05, **Figure 4C**). Following treatment with TGFβ1, there was a main effect of treatment for pmTOR/mTOR (P < 0.05, **Figure 4A**), pRaptor/Raptor (P < 0.05, **Figure 4B**) and pRictor/Rictor (P < 0.01, **Figure 4C**) but post-hoc analysis revealed no differences between TGFβ1 doses (P > 0.05).

### Glucose Transport

**Figure 5:**
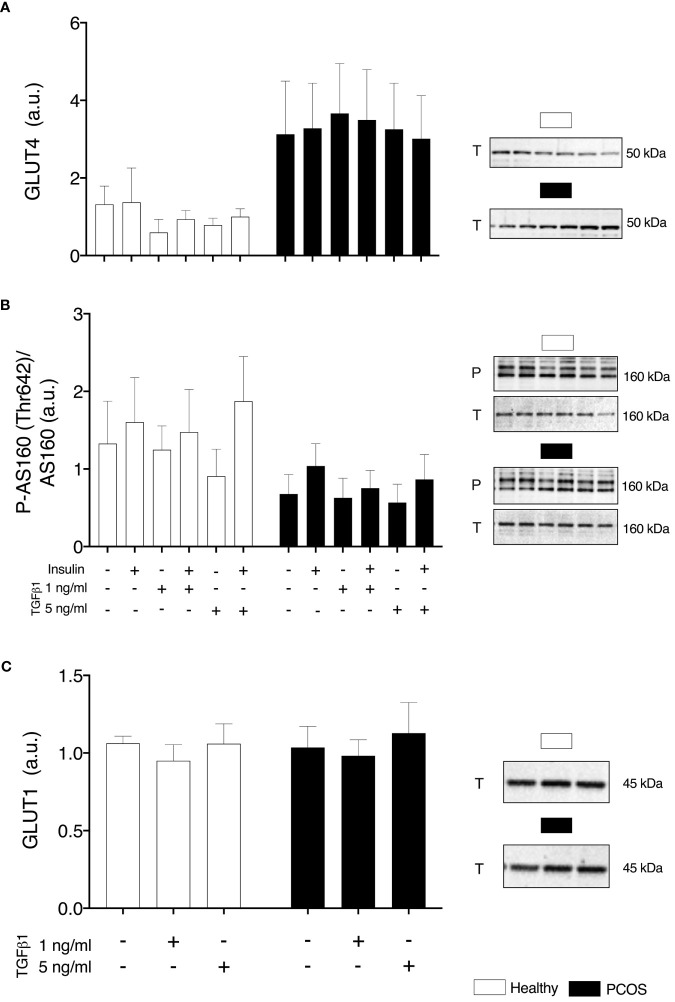
Glucose Transport. **(A)** Total GLUT4 expression. **(B)** AS160 phosphorylation relative to total AS160 expression, **(C)** Total GLUT1 expression, following 16 h with no treatment or with TGFβ1 (1 ng/ml or 5 ng/ml) and 30 min insulin stimulation (0 nM or 100 nM). Data are reported as Mean ± SEM. AU, arbitrary units (defined as band density values). Phospho (P) and Total (T). Healthy: N = 4-5 participants (clear bars), PCOS: N= 4-5 participants (filled bars).

In order to determine if any intrinsic defects were present in glucose transport or were induced by TGFβ1, we assessed GLUT1 and GLUT4 content and phosphorylation of AS160^Thr162^. We found no differences in GLUT4 (P > 0.05, **Figure 5A**), pAS160/AS160 (P > 0.05, **Figure 5B**) or GLUT1 (P > 0.05, **Figure 5C**) between groups or TGFβ1 doses.

### Extracellular Matrix

**Figure 6:**
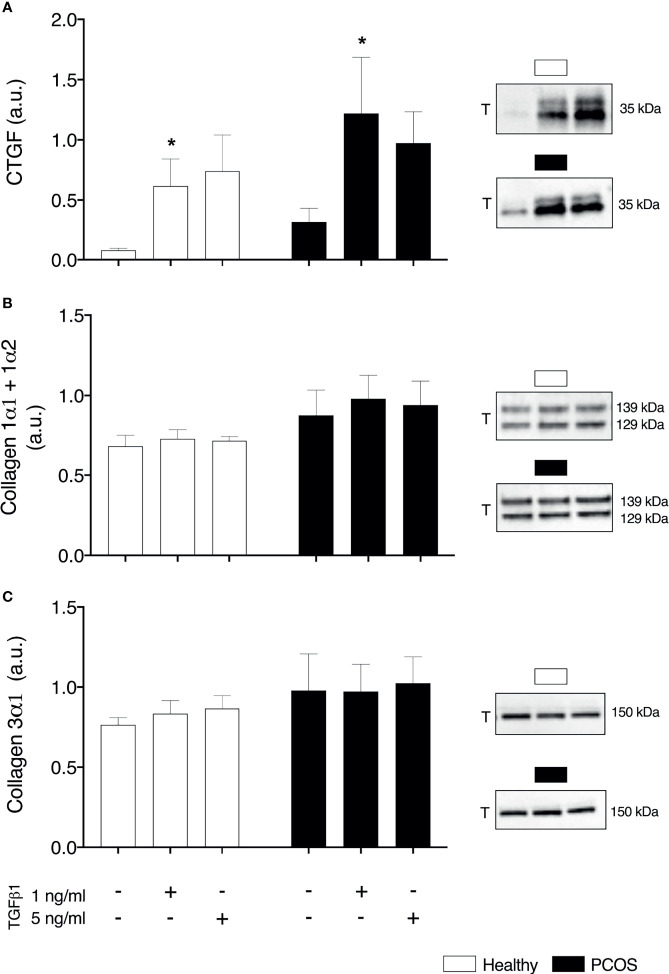
Extracellular matrix. **(A)** Total Connective tissue growth factor (CTGF/CCN2) expression **(B)** Total collagen 1α1 and 1α2 **(C)** Total collagen 3α1. *significantly different from untreated control of the respective group (P < 0.05). Total (T). Data reported as Mean ± SEM. AU, arbitrary units (defined as band density values). Healthy: N = 5 participants (clear bars), PCOS: N= 5 participants (filled bars).

To examine whether TGFβ1 could contribute indirectly to insulin resistance by the accumulation of the ECM, we assessed pro-fibrotic factor CTGF and collagen1α1; 1α2 and 3α1 which account for a large percentage of the ECM. We identified a main effect of group in the basal expression of CTGF (P < 0.05, **Figure 5A**), but post-hoc analysis revealed no differences between groups (P > 0.05, **Figure 6A**). Following treatment, there was an increase in basal expression of CTGF following TGFβ1 1 ng/ml treatment in both myotubes from healthy women (P < 0.05, **Figure 6A**) and from women with PCOS (P < 0.05, **Figure 6A**). There were no differences in Collagen1α1; 1α2 and 3α1 between groups (P > 0.05, **Figures 6B, C**) or TGFβ1 doses (P > 0.05, **Figures 6B, C**).

## Discussion

In this study, we aimed to determine whether TGFβ1 is in part responsible for the development of insulin resistance and/or aberrant insulin signalling, previously observed in the skeletal muscle and myotubes of women with PCOS. From the clinical characteristics, women with PCOS display profound insulin resistance and hyperandrogenism in comparison to healthy women. This was demonstrated *via* elevated fasting insulin and free testosterone coupled with a reduced insulin sensitivity as measured by euglycaemic–hyperinsulinaemic clamp. These findings are consistent with previous work from our lab and others (5, 6, 60). At baseline, myotubes from women with PCOS and healthy women displayed similar levels of glucose uptake and expression of insulin signalling proteins. The lack of differences observed between groups in glucose uptake and insulin signalling in the untreated myotubes, suggests that myotubes from women with PCOS do not retain their metabolic donor characteristics. This is in line with previous glucose uptake, glycogen synthesis and mitochondrial function studies showing no differences between myotubes from women with PCOS and matched controls (8, 12, 13). Therefore, our study supports the view that an intrinsic defect in skeletal muscle is unlikely to be responsible for *in vivo* insulin resistance in women with PCOS. Myotubes established from women who are severely obese (BMI >40 kg/m^2^) have been demonstrated to have an altered glucose metabolism, displaying increased reliance on glycolysis compared to myotubes from women who are lean (61, 62), with these metabolic defects in myotubes being reversed following gastric bypass surgery (63). Signalling defects such as a reduction in insulin-stimulated phosphorylation of AKT and increase in basal and insulin-stimulated phosphorylation of IRS-1_ser312_ have also been observed in myotubes derived from obese individuals (64, 65). In the case of our results, we did not see a clear effect of obesity on glucose metabolism or insulin signalling, reinforcing that skeletal muscle insulin resistance in women with PCOS is not a result of an intrinsic defect.

Based upon its role in the pathophysiology of PCOS and preliminary evidence (16, 33–35), we attempted to establish if TGFβ1 is a circulating factor that could contribute to the development of skeletal muscle insulin resistance. Contrary to our hypothesis, treatment with TGFβ1 resulted in an increase in glucose uptake in myotubes from women with PCOS and healthy women. This increase occurred following a lower dose (1 ng/ml) in the myotubes from healthy women and following the higher dose (5 ng/ml) in the myotubes from women with PCOS. This difference presents the possibility that myotubes from PCOS are desensitised to the effects of TGFβ1 as they are chronically exposed to higher levels and may require a more potent stimulus to respond. It could be hypothesised that the myotubes from the women with PCOS exhibit a memory effect to *in vivo* exposure to TGFβ1, similar effects have been shown for another cytokine: TNFα (66). Although TGFβ1 has been suggested to induce glycolysis and increase expression of GLUT1 *via* SMAD2/3 in other cell types (42, 45, 67), the increase in glucose uptake occurred independently of changes in protein expression of glucose transporters: GLUT1 and GLUT4. Similar responses in myotubes and other cell types have been shown following stimulation with cytokines or hormones, where an increase in glucose uptake *via* GLUT1/4 translocation occurs independently of changes in protein expression (68–70). This could explain the increase in glucose uptake with no changes in protein abundance of glucose transporters observed in our study, as we have not specifically assessed changes in translocation rates. We recommend future studies to assess the effects of TGFβ1 on glucose transporter translocation to understand how TGFβ1 can increase glucose uptake rather than measuring GLUT protein expression alone.

We also observed that TGFβ1 increased phosphorylation of SMAD3 and did not significantly activate SMAD1/5/9, as expected. Insulin can prompt rapid translocation of intracellular TGFβ receptors 1 and 2 to the cell surface in various cell types (36, 37). This occurs *via* the activation of Akt and is regulated by subsequent activation of AS160 to enhance TGFβ responsiveness (36). This translocation of TGFβ receptors causes an amplification of TGFβ signalling through SMAD activation (38), presenting the possibility of insulin enhancing TGFβ signalling. However, in our study, we did not see any additive effect of insulin on SMAD signalling or interaction that could influence insulin signalling. Previous results from C2C12 myotubes suggest that TGFβ1 treatment results in increased phosphorylation of SMAD3, which in turn can suppress insulin-stimulated phosphorylation of AKT and AS160, as well as reduce expression of GLUT4 (71, 72). To date, only one other study using human myotubes has assessed the metabolic signalling following treatment with 1 ng/ml TGFβ1 (73), which demonstrated a decrease in gene expression of mitochondrial regulators and slight suppression of insulin stimulated AKT phosphorylation (73). The precise reasons behind the differences in results is not clear. This could be related to sex-specific effects, however, the authors (73) did not report the participant characteristics from which skeletal muscle cell lines were established.

In contrast to these results, our study showed that TGFβ1 increased glucose uptake but had no significant inhibitory effect on insulin signalling proteins despite increasing phosphorylation of SMAD3. The contrasting response with our results with human primary myotubes, and studies from C2C12 myotubes, may be explained by differences in structure (e.g. differences in gene expression of myosin heavy chains and ECM), metabolic behaviours (e.g. insulin responsiveness, basal glucose uptake, and gene expression of glucose transporters) and donor variability (74). Another critical difference between our study and previous studies is the length of time the TGFβ1 treatment was applied to the cells. We opted for 16 hours of treatment, whereas others opted for a shorter transient treatment from 30 minutes to 3hours (71, 72).

One of the mechanisms by which TGFβ1 and associated signalling has been proposed to contribute to the development of insulin resistance is by ECM remodelling. This occurs by increased collagen deposition in the endomysium, epimysium and basement membrane, creating a physical barrier (29, 32, 72, 75, 76). We observed no changes in collagens types I and III following treatment with TGFβ1, although we did observe an increase in pro-fibrotic CTGF in myotubes from both groups with a dose of 1 ng/ml. The lack of changes in collagens may be a time-course issue, as observed with changes in the expression of collagens and laminin β1 following muscle-damaging exercise or electrical stimulation being absent after 2 days but present after 27-30 days (77, 78). Similarly, changes in skeletal muscle collagens (I and III) and ECM structure were absent three months after gastric bypass in individuals with type 2 diabetes, but present after 9 months (72). Most of the studies have investigated fibrosis/ECM remodelling in skeletal muscle tissue, which includes various cell types; however, it has been shown that myotubes and myofibers are capable of producing collagens in the absence of fibroblasts (79). Indeed, when mature myotubes treated with exogenous SPARC (a protein that induces collagen production and influences ECM assembly), they display an increase in collagen1α1 protein expression (80). However, to date, there is a lack of studies showing an increase in protein expression of collagens in skeletal muscle myotubes, with the majority measuring gene expression only. CTGF is overexpressed in skeletal muscle of individuals with Duchenne muscular dystrophy (21) and has been shown to interact with TGFβ1 to produce an increase in the expression of ECM proteins (20, 23). Furthermore, it has been demonstrated that CTGF is required for TGFβ1 to induce increases in pro-fibrotic genes in C2C12 myotubes (22). This would suggest that the increase in CTGF expression with TGFβ1 we observed may be a precursor of fibrosis, although it was not sufficient to cause any insulin signalling dysregulation in the myotubes. Further studies are required to investigate the impact of a longer-term TGFβ1 exposure in myotubes that may induce a sustained increased expression of this fibrosis precursor and promote adverse ECM remodelling.

There are a number of limitations with the current study, the use of western blotting to assess insulin and TGFβ signalling only allows a limited number of targets to be analysed. Therefore, aspects of their signalling not analysed in this current study may have provided further insight into the relationship between the two signalling pathways. Furthermore, we only assessed insulin signalling following 30 minutes of insulin stimulation, which means that we may have missed differences in acute phosphorylation events between groups or treatments as previously demonstrated (81, 82). In addition, given the vast number of phosphorylation sites for proteins involved in insulin signalling, we cannot rule out an effect of the treatment or disease state on other sites that were not measured in our study. We also selected a supraphysiological dose of insulin (100 nM), which was based on the majority of previous primary myotube studies from women with PCOS focusing on insulin resistance (8, 9, 13, 83). Whether lower doses of insulin treatment may have altered the responses we observed is not clear. Furthermore, while a sample size of seven participants per group is an acceptable number for studies involving invasive muscle biopsies and the derived human primary cell culture work, future large-scale studies are needed to confirm our findings and clarify further the role of TGFβ1 in glucose metabolism and insulin signalling.

## Conclusion

In conclusion, TGFβ1 treatment in myotubes increased glucose uptake, suggesting that short term increased serum TGFβ1 or localised TGFβ signalling dysfunction are unlikely to induce insulin resistance *via* defects in insulin signalling in the skeletal muscle. However, TGFβ1 treatment in myotubes derived from women with or without PCOS also promoted the expression of SMAD3 and CTGF. This may suggest that chronic exposure to elevated levels of TGFβ1, such as that present in women with PCOS, could induce a pro-fibrotic phenotype and ECM remodelling, which may consequently impede insulin signal transduction. Further *in vivo* studies are required to investigate the effect of acute and chronic TGFβ1 exposure on indirect insulin resistance mechanisms, such as mitochondrial dysfunction (73, 84); and the relationship between ECM composition and structure with insulin resistance in skeletal muscle of women with PCOS.

## Data Availability Statement

The raw data supporting the conclusions of this article will be made available by the authors, without undue reservation.

## Ethics Statement

The studies involving human participants were reviewed and approved by Ethical approval was obtained from the Victoria University Human Research Ethics Committee (Reference - HRE17-232). The patients/participants provided their written informed consent to participate in this study.

## Author Contributions

AM-A, AM, NS, and RR were responsible for the conception of the study and funding acquisition. LM, RP, NS, and AM-A carried *in vivo* and *in vitro* data collection and experiments. LM and AM-A carried out the data analysis and drafted the manuscript. RP, AM, and RR reviewed and edited the manuscript. All authors approve the submitted version to be published and agree to be accountable for all aspects of the work.

## Funding

This work was supported by Diabetes Australia Research Project Grant [Grant number: Y18G-STEN] awarded to NS, AM-A, AM, and RR. Funding source had no involvement in the conceptualisation, data collection or publication.

## Conflict of Interest

The authors declare that the research was conducted in the absence of any commercial or financial relationships that could be construed as a potential conflict of interest.

## Publisher’s Note

All claims expressed in this article are solely those of the authors and do not necessarily represent those of their affiliated organizations, or those of the publisher, the editors and the reviewers. Any product that may be evaluated in this article, or claim that may be made by its manufacturer, is not guaranteed or endorsed by the publisher.

## Abbreviations

CTGF, Connective Tissue Growth Factor; ECM, extracellular matrix; PCOS, Polycystic Ovary syndrome; TGFβ, Transforming Growth factor-beta; [2-^3^ H]DG, [2-^3^H]deoxy-D-glucose.
